# Micro-Organisms in Water

**Published:** 1884-03

**Authors:** 


### Micro-organisms in Water.

Osmic acid possesses the property of hardening protoplasm, hence microscopists make use of it for detecting animalcula in water. According to L. Maggi, the chloride of palladium may be substituted for the more poisonous osmic acid, an acid more dangerous than prussic acid. By adding to the water a solution of palladium chloride (one in eight hundred) he obtained a precipitate in which the bacterial forms could be recognized under the microscope just as distinctly as in those obtained with osmic acid.—*Gazz. Chim.*

